# A Real-Time Artificial Intelligence-Assisted System to Predict Weaning from Ventilator Immediately after Lung Resection Surgery

**DOI:** 10.3390/ijerph18052713

**Published:** 2021-03-08

**Authors:** Ying-Jen Chang, Kuo-Chuan Hung, Li-Kai Wang, Chia-Hung Yu, Chao-Kun Chen, Hung-Tze Tay, Jhi-Joung Wang, Chung-Feng Liu

**Affiliations:** 1 Department of Anesthesiology, Chi Mei Medical Center, Tainan 710, Taiwan; 0201day@yahoo.com.tw (Y.-J.C.); ed102605@gmail.com (K.-C.H.); anesth@gmail.com (L.-K.W.); dkntstar@hotmail.com (C.-H.Y.); 400002@mail.chimei.org.tw (J.-J.W.); 2 College of Health Sciences, Chang Jung Christian University, Tainan 710, Taiwan; 3 General Education Center, Chia Nan University of Pharmacy and Science, Tainan 717, Taiwan; 4 Department of Thoracic Surgery, Chi Mei Medical Center, Tainan 710, Taiwan; a.kun.ke@gmail.com; 5 Department of Intensive Care Medicine, Chi Mei Medical Center, Tainan 710, Taiwan; milkmaid29@yahoo.com; 6 Department of Medical Research, Chi Mei Medical Center, Tainan 710, Taiwan; 7 Center for Big Medical Data and Artificial Intelligence Computing, Department of Medical Research, Chi Mei Medical Center, Tainan 710, Taiwan

**Keywords:** lung resection, pulmonary function test, artificial intelligence, machine learning, pre-anesthetic consultation, staged weaning

## Abstract

Assessment of risk before lung resection surgery can provide anesthesiologists with information about whether a patient can be weaned from the ventilator immediately after surgery. However, it is difficult for anesthesiologists to perform a complete integrated risk assessment in a time-limited pre-anesthetic clinic. We retrospectively collected the electronic medical records of 709 patients who underwent lung resection between 1 January 2017 and 31 July 2019. We used the obtained data to construct an artificial intelligence (AI) prediction model with seven supervised machine learning algorithms to predict whether patients could be weaned immediately after lung resection surgery. The AI model with Naïve Bayes Classifier algorithm had the best testing result and was therefore used to develop an application to evaluate risk based on patients’ previous medical data, to assist anesthesiologists, and to predict patient outcomes in pre-anesthetic clinics. The individualization and digitalization characteristics of this AI application could improve the effectiveness of risk explanations and physician–patient communication to achieve better patient comprehension.

## 1. Introduction

Lung cancer has the highest mortality rate among all cancers in Taiwan [1]. The standard treatment for lung cancer is tumor resection surgery before cancer cell metastasis. However, lung cancer is most often detected in the metastatic stage [2]. Therefore, only a small percentage of patients with lung cancer can undergo surgery.

Lung resection is one of the riskiest major surgeries and must be performed under general anesthesia with advanced anesthetic monitoring [3]. Patients undergoing lung resection surgery must receive endotracheal tube intubation, which provides access to mechanical ventilation via a machine called a ventilator. The ventilator helps patients achieve optimal oxygenation during general anesthesia. If a patient can be weaned from the support of the endotracheal tube and the mechanical ventilator immediately after lung resection surgery (referred below as “wean immediately”), they can be sent back to the general ward for postoperative care; otherwise, they should be transferred to the intensive care unit (ICU) for temporal support with mechanical ventilation via an endotracheal tube, and ventilatory support should be gradually withdrawn, usually within days of surgery, based on clinical respiratory evaluations (referred to below as “staged weaning”) [4,5,6,7].

All patients undergoing lung resection surgery routinely visit the pre-anesthetic consulting clinic for risk evaluation, explanation, and discussion with the anesthesiologists before general anesthesia. The possibility of transferring to the ICU postoperatively for staged weaning and the possibility of difficult weaning is one of the most important topics in the pre-anesthetic consulting clinic.

Well-known factors related to staged weaning for lung surgery are [6] (p. 1945–1953), as follows: (1) postoperative residual lung function, usually estimated using the postoperative forced expiratory volume in one second (ppoFEV1); the ppoFEV1 is calculated by pre-operative FEV1% × (1 % lung volume resected during operation/100) [6] (p. 1944); generally, ppoFEV1% >40% represents a low risk factor for respiratory complications [6] (p. 1945) [7]. (2) Detailed history of smoking and quitting smoking; smokers who ceased smoking for more than four weeks before thoracic surgery had a lower rate of pulmonary complications than those who did not [6] (p. 1949) [8]. (3) Resting oxygen saturation before surgery. (4) The maximum exercise loads patients can afford before operation, for example, whether they can climb more than two floors without resting in between, and whether they can walk for over six minutes. (5) Severity of coronary atherosclerosis or coronary artery disease (CAD). The coronary arteries supply oxygen and blood to the heart muscles. Deoxygenation of the heart muscle can cause myocardial infarction, irregular rhythms, or heart failure; those who have any of the above-mentioned conditions require high concentration oxygen treatment and staged weaning in the ICU. (6) Major medical diseases such as liver failure, renal failure, and morbid obesity (body mass index ≥ 35 kg/m^2^), or neuromuscular diseases that causes weakness, such as myasthenia gravis. (7) Current age [6,9] (p. 1947) (8) The reversal agents of the muscle relaxants allow the airway muscles to contract with full strength and prevent postoperative residual curarization; therefore, a patient can breathe without ventilator support after emergence from general anesthesia [10,11,12,13].

Each of the above-mentioned weaning factors has its own thresholds [14], has its own contributions to staged weaning, and has been investigated separately in previous studies. No study has yet investigated the contribution of each risk factor for staged weaning, the integrated risk assessment, the relevance of each potential risk factor, or their impact; nor have any previous studies provided an appropriate comprehensive predictive model in the context of “cannot wean immediately” after lung resection surgery.

Using individualized risk factors for risk prediction is a future trend in precision medicine [15]. The use of artificial intelligence (AI)-supervised machine learning-based techniques provides a feasible solution. This study aimed to use the nine factors mentioned above to generate a computerized AI prediction model that would be useful for objective pre-anesthetic evaluation.

It is expected that the application of this model could aid in the following: (1) estimation of the potential requirements of “cannot wean immediately” after lung resection surgery, with data that facilitates understanding by patients and their family members in the pre-anesthetic evaluation clinic; (2) personalized and customized data, especially for patients presenting a poor pre-operative condition, potentially helping the patient and their family in setting realistic expectations to help prevent medical disputes; and (3) to provide different predictive data for the use of different types of anesthesia reversal agents [16,17,18].

AI approaches are a promising strategy for achieving individualized digitalization of weaning possibility prediction. A better AI-assisted prediction model can improve patients’ comprehension and physician–patient communication.

This paper consists of introduction, material and methods, results, discussion, and conclusion sections. The introduction section captures the purpose of this study, which was to analyze the impact of the nine mentioned well-known factors affecting weaning possibility. The secondary aim was to build a computerized AI prediction model application. It was hypothesized that previous medical big data could provide accurate future predictions. The goal of the present study would be of particular interest to the presentation on applying the AI application for physicians, and to obtain a better understanding of weaning possibility in patients. The material and methods section describes step by step the investigation of the previous medical big data on lung resection surgery in Chi Mei medical center and attempts to input the big data into an AI algorithm for future predictions. The results section consists of the verified AI algorithms by model testing and shows the details of the testing report. More specifically, we tested the AI prediction models with different AI algorithms to obtain the best model for application. The discussion section presents the advantages of AI prediction models, the comparisons between AI and traditional mathematical equations to predict prognosis, the limitations, and our future goals and possibilities of future research. The conclusion section consists of a summary and practice implications.

## 2. Materials and Methods

This was a retrospective study. After institutional review board approval, we collected the data of patients who underwent thoracotomy for lung tumor resection at Chi Mei Medical Center between 1 January 2017 and 31 July 2019. In total, 709 patients underwent surgery, and patients under 20 years of age were excluded. We deleted the medical record number and all types of personal identification of each patient and randomly assigned a new code to each patient. This encryption method is used to protect patient privacy. The present study was approved by the Institutional Review Board of the Chi Mei Medical Center (IRB Serial No.: 10810-001).

According to the literature [6], data on the following factors were collected for each patient as feature variables: (1) pre-operative lung-related test data, (2) previous heart-related reports and history of cardiovascular surgery, (3) volume of lung tissue expected to be resected during surgery, (4) maximum exercise load, (5) current balance of oxygen supply and demand, (6) other important medical history that could affect intensive care unit admission; (7) history of smoking and smoking cessation; (8) current age; and (9) muscle relaxant reversal agent used (sugammadex or neostigmine) [10,11,12,13]. Pre-operative lung function data and the volume of lung tissue expected to be resected were combined to calculate the predicted postoperative residual lung function, which was represented by the predicted ppoFEV1.

All data of the above-mentioned nine factors were used as feature values (X value), and whether the patients were weaned immediately after operation was used as the outcome value (Y value). The threshold for binary classification of the X values was set as described in the literature, that is, the cutoff point between normal and abnormal values binary classifying the features based on the most authoritative textbook of anesthesiology [6] (Supplementary Material—Table S1).

We used Spearman correlation analysis (heat map) [19] to investigate the contribution of each feature to the outcome. All the differences between groups were calculated using T-test and predetermined analysis with a significance level of *p* < 0.001 (two-sided) using SPSS (Statistical Product and Service Solutions) software (version 19.0, Chicago II., IBM Corp, Armonk, NY, USA).

After encryption, the data were imported into a machine-learning prediction model programmed in Python. Various predictive models were constructed using Python programs, hereinafter referred to as model construction.

For model construction, a random selection method was used, in which 70% of the patient data were randomly selected for AI model training, and 30% of the patient data were used for model testing. To balance the dataset with an imbalanced outcome class, the synthetic minority over-sampling technique (SMOTE) [20,21,22,23,24,25,26,27] was applied. SMOTE is an oversampling technique in which the minority class is oversampled by creating “synthetic” examples along the line segments joining any or all of the minority class nearest neighbors. The SMOTE technique was adopted only for the training dataset.

We used multiple supervised machine learning AI algorithms, including logistic regression, random forest, support vector machine (SVM) [28,29], light gradient boosting machine (light GBM) [30], multilayer perceptron (MLP) [31], extreme gradient boosting (XGBoost) [31], and Naïve Bayes Classifier [32,33,34,35]. We used Python version 3.7.6, scikit.learn version 0.22.2.post1, Tensorflow:version 2.1.0, conda: version 4.8.3, Keras 2.3.1, Numpy 1.18.1, Pandas 1.0.3, imbalanced-learn 0.6.2, Light GBM 2.3.1, XGBoost 1.1.1, and matplotlib 3.1.3 in our development platform. The full details of the selected hyperparameters for each algorithm are listed in tabular form (Supplementary Material—Table S2). The modeling results were measured by accuracy, sensitivity, specificity, positive predictive value, negative predictive value, receiver operating characteristic curve (ROC), and area under the receiver operating characteristic curve (AUC) [36]. We compared the performance of the seven AUCs between algorithms [28,32,37,38,39,40].

## 3. Results

### 3.1. Study Period and Case Number

A total of 709 patients who underwent lung resection surgery at Chi Mei Medical Center between 1 January 2017 and 31 July 2019 were recruited for the present study.

#### 3.1.1. Patient Grouping According to Outcome

Patients were divided into two groups according to whether they underwent stage weaning or not: 555 patients were weaned from the endotracheal tube immediately after the operation, and 154 patients failed to wean immediately and were transferred to the ICU for stage weaning.

#### 3.1.2. Detailed Baseline Characteristics

The detailed baseline characteristics and grouping according to the outcome are shown in Table 1.

**Figure 1:**
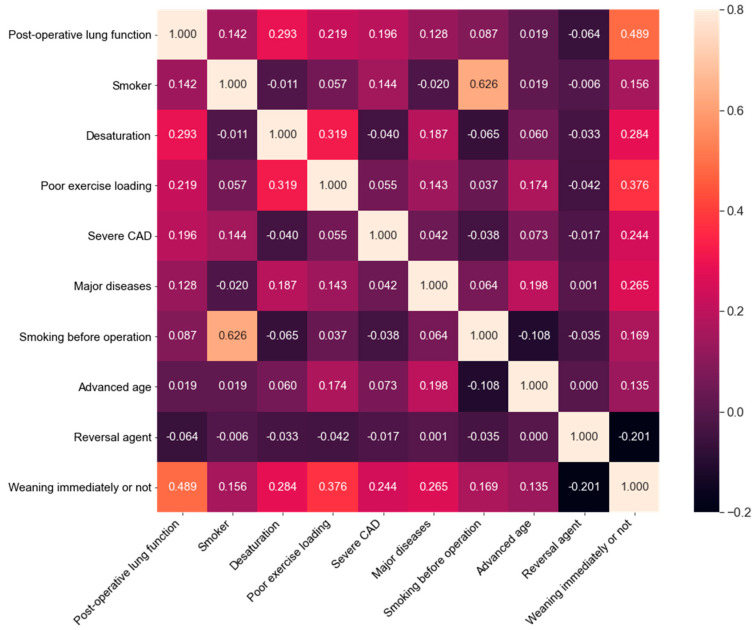
Spearman correlation analysis (Heat map).

The Spearman correlation analysis (heat map) (Figure 1) presents the contribution of each feature to the outcome. Estimated post-OP lung function and exercise loading had the strongest positive correlation with outcome. The outcome correlation from the strongest to the weakest was as follows: estimated post-OP lung function > exercise loading > resting oxygen saturation before operation/desaturation or not > major diseases > severe CAD > reversal agent > quit smoking or not before operation > presence of smoking history > advanced age. In heat map, absolute value is much more important than positive value or negative. The negative value of reversal agent feature is because of our study settings, we set all the abnormal data as 1, and the normal, healthier data as 0, only except for reversal agent medication: the usage of sugammadex, which is better medication for preventing postoperative residual curarization, as 1. Detail of binary classifying settings please see Table S1.

### 3.2. AI Intervention

#### 3.2.1. AI Algorithms

We used seven AI machine learning algorithms–logistic regression, random forest, SVM, light GBM, MLP, XGBoost, and Naïve Bayes Classifier to build prediction models. Grid search with 5-fold cross-validation for hyper-parameters tuning for each algorithm was conducted to obtain the optimal model (the hyper-parameters are summarized in Table S2, Hyper-parameters range for experiments). In total, 709 cases were used for outcome modeling with 70% as training dataset and 30% as testing dataset.

#### 3.2.2. Testing Results of Prediction Models

**Figure 2:**
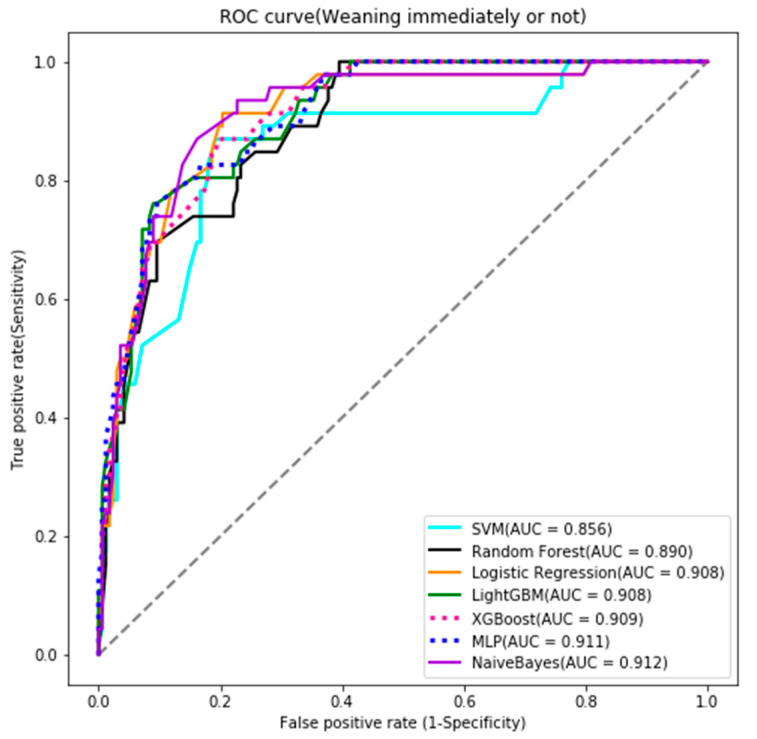
Receiver operating characteristic curve (ROC) of testing result.

The prediction models were tested and measured by accuracy, sensitivity, specificity, positive predictive value, negative predictive value, and AUC (Table 2). Compared with other algorithms, the Naïve Bayes Classifier algorithm had the best testing result (Table 2) and the best performance in the ROC curve. Validation of the AI prediction model with new patients also showed good accuracy (Figure 2).

### 3.3. Clinical Application

#### Embedment of the above AI Prediction Model in the Pre-Anesthetic Clinic

The Naïve Bayes Classifier prediction model showed the best testing results among all the AI-assisted prediction models and was therefore selected for subsequent clinical applications. We also assessed whether each naive Bayes classifier prediction had sufficient reproducibility, that is, whether the constructed model was stable enough that the prediction results did not differ too much each time. After confirming that the prediction had sufficient reproducibility, the comprehensive machine learning prediction constructed using NB was considered to have achieved a certain level of accuracy, and the model was deemed stable, reliable, and capable of providing anesthesiologists with a clinical predictive reference to support clinical services.

**Figure 3:**
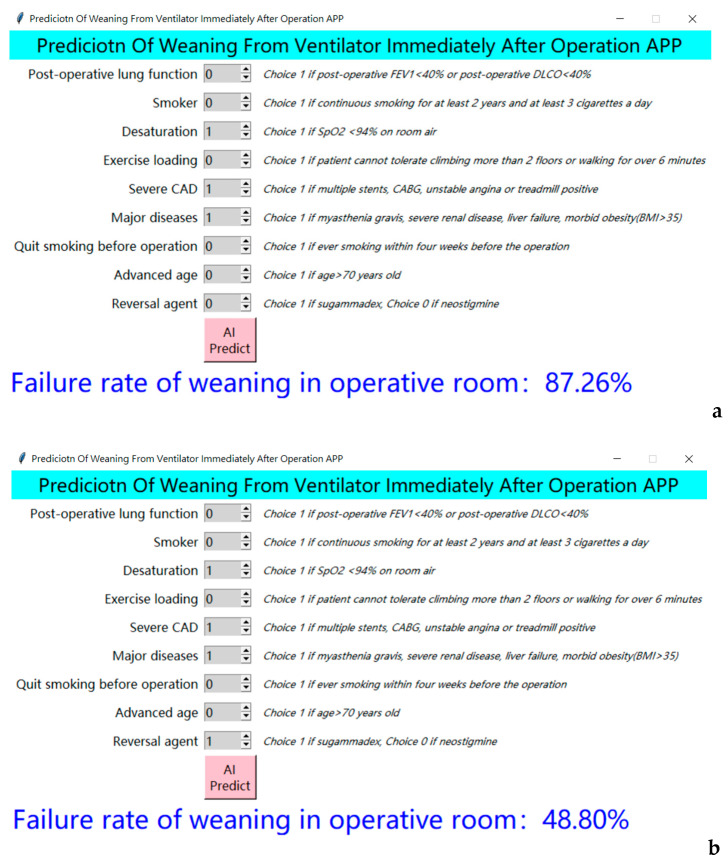
Final application software of the AI predicting system, implanted in the computer of the preanesthetic clinic. (**a**) Screenshots of AI predicting system applied to a patient receiving neostigmine. (**b**) Screenshots of AI predicting system applied to a patient receiving sugammadex.

After a stable and reliable model of NB was constructed, we cooperated with the AI center and computer engineers to embed the above AI prediction model in the pre-anesthetic evaluation computer screen. We implemented the best model as a friendly application (app) to assist physicians in clinical decision-making and communication with patients (Figure 3).

### 3.4. Satisfaction Score, before and after AI Application

We also calculated the satisfaction score and compared the scores between the traditional and AI groups.

For Anesthesiologists

The anesthesiologists were divided into two groups based on their working seniority:

- Junior anesthesiologists (with less than 10 years of experience)
- Senior anesthesiologists (who had more than 10 years of clinical experience)

#### 3.4.1. The Subjective Benefit Scores

Anesthesiologists were given the question: “Does the AI system help you in the consulting clinic?”, and they answered with a score based on the following five-point ordered response scale: 1 = strongly disagree, 2 = disagree, 3 = neither agree nor disagree, 4 = agree, and 5 = strongly agree, which was viewed as a subjective benefit score. The subjective benefit score can be used as a presentation of user satisfaction to some degree.

The subjective benefit scores of the anesthesiologists were high. Both junior and senior anesthesiologists recorded high benefit scores for this AI system (average subjective benefit score: junior vs. senior anesthesiologists, 5 vs. 4.57, *p* = 0.0537).

#### 3.4.2. Subjective Perception of Time-Saving Percentage

**Figure 4:**
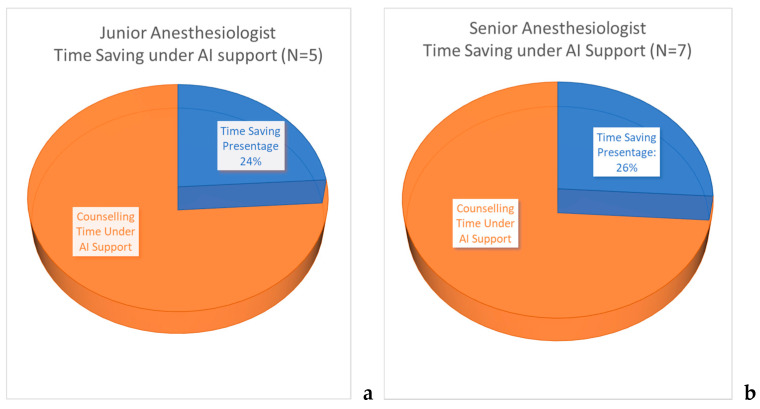
Time-saving percentage after using the AI system among anesthesiologists working in preanesthetic consulting clinic. (**a**) The subjective perception of junior anesthesiologists. (**b**) The subjective perception of senior anesthesiologists.

Anesthesiologists recorded the percentage of time saved after AI system implantation, compared to that before AI assistance. The entire circle (360 °, 100%, Figure 4) indicates the time spent on each consultation. The blue area represents the percentage of time saved due to AI assistance for each consultation. Both junior and senior anesthesiologists felt that the AI predictive system provided some degree of support in saving time (24% for junior anesthesiologists and 26% for senior anesthesiologists; *p* = 0.4363) (Figure 4).

#### 3.4.3. Patient Satisfaction Scores

**Figure 5:**
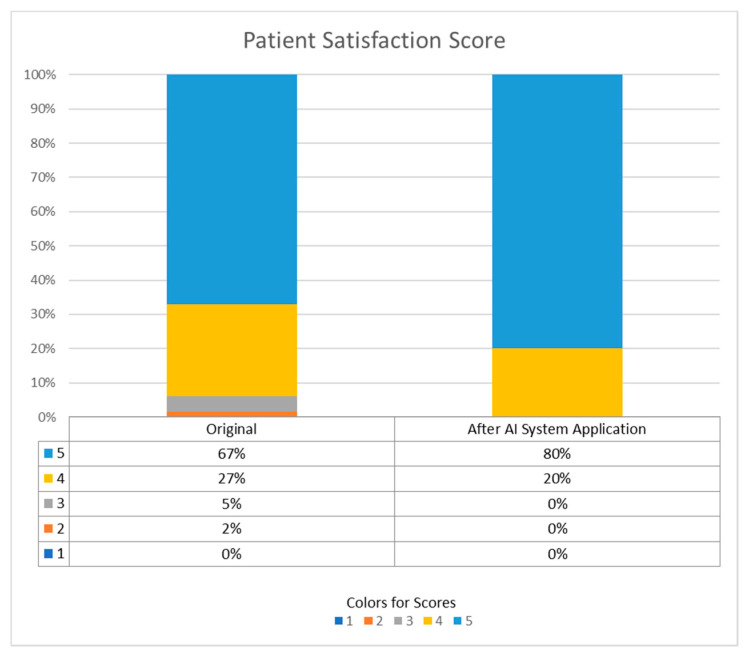
Patient satisfaction score for the understanding of anesthetic explanation, before and after AI system implantation.

Patient satisfaction scores were routinely evaluated in our pre-anesthetic clinic for several years. Patients or their key family were given the question, “Are you satisfied with the risk explanation in the consulting clinic? Do you understand the anesthetic explanation?”, and they answered with a score based on the following five-point ordered response scale: 1 = strongly disagree, 2 = disagree, 3 = neither agree nor disagree, 4 = agree, and 5 = strongly agree, which was viewed as the satisfaction score. We compared the patient satisfaction scores before and after AI system implantation, as the “original group” and the “after AI system application group” We labeled the scores in different colors in Figure 5. The decrease of scoring less than 5 after AI system application can be used as a representation of higher satisfaction.

In the AI system application group, 50% of patients strongly agreed (5 points) and 50% agreed (4 points) that digitalization improved their understanding of their risk of inability to wean immediately, the demand for high concentration oxygenation from the ventilator, and the related anesthetic risk.

## 4. Discussion

The comprehensive digitalization of medical data has been a trend in recent years [41]. Digitization helps clinicians quickly access a large amount of historical medical data. After digitalization is completed, it provides a platform for further investigation [42], that is, trying to use medical big data to provide accurate future predictions. The more accurate the prediction, the more clinicians can put resources into key areas for better resource allocation. Without performing more invasive medical interventions, making predictions using past data alone requires the introduction of new computer technology [43]. AI can be the best helper in such areas and can lead to better efficiency.

AI prediction models can help in performing overall integrated risk assessment [19]. If the prediction model has sufficient accuracy, predictive power, and high reproducibility for each prediction, it can assist clinical physicians in providing individualized risk evaluations based on previous medical records. Anesthesiologists are responsible for providing patients and their families with operative risk evaluation in the pre-anesthetic clinic. However, the weight of each possible factor has not been clearly defined in previous studies. Moreover, patients diagnosed with lung cancer often have comorbid diseases. Factors that independently influence muscle power, such as neuromuscular disorders or muscular reversal agents, should be considered simultaneously. Our AI system can link the historical data of the Chi Mei Medical Center and maintain manual input by the clinician to adapt to the patient’s medical records from other hospitals that are not captured in the Chi Mei medical center. Because the input can be adjusted manually at any time, the prediction result can be updated immediately, for example when the patient’s blood oxygen changes to respond to the patient’s current blood oxygen state. Maintaining manual input for adjustments makes our prediction system flexible and convenient for users. According to a questionnaire survey of users, both anesthesiologists and patients were satisfied with the AI-assisted prediction system. Our AI-based prediction system can help anesthesiologists in integrated risk assessment for all risks in a pre-anesthetic clinic. Our results show that digitalization by an AI-assisted prediction system increases the efficiency of pre-anesthetic consultation. Anesthesiologists feel that they have benefits in improving the consultation. It can also save anesthesiologists’ time in pre-anesthetic consulting clinics. Digitalization by the prediction system also improves the understanding of patient risk. Our results also showed that the more patients understood, the more satisfied they were with the information gained in the pre-anesthetic counseling clinic.

Individualized medicine and precision medicine are trends that will dominate in the future. Individualization can enhance shared decision-making quality before patients make major medical decisions [16,17,44,45]. Since our prediction system is personalized based on pre-operative data, it may improve the acceptance and comprehension of patients and their families. Understanding and acceptance will eliminate the potential disparity between patient expectations and reality and might reduce medical disputes.

The use of previous medical big data, AI, and medical technology to construct medical predictive models is important for the future of medicine [19,41,46,47]. Most previous studies have calculated each risk as an independent variable in mathematical equations, trying to use these to predict prognosis. Unfortunately, processing data using mathematical equations, without the assistance of artificial intelligence, poses three major problems [41]: first, the more variables in a mathematical equation and the more complex the formula, and the more difficult it is to quickly calculate with the human brain [15]. Second, once the equation is completed, any modification, even the expansion of more data, may destroy the original structure. Modifications will require too much time, such that mathematical equations are considered unsuitable for dynamic adjustments. Third, the prognostic outcome of previous studies is often present as risk stratification, which is not intuitive enough for patients and their family members. The advantage of big data and AI is that, after the model is completed, new data can be used as a test of the previous model, and at the same time as an expansion for the database [15]. The longer the AI program runs, the more accurate the prediction, and the remaining real-time output of results.

Among our training algorithms, the Naïve Bayes Classifier is the best, with the highest AUC and a more balanced sensitivity and specificity. The Naïve Bayes Classifier is one of the most efficient and effective inductive algorithms for classification, in which all attributes are independent, given the value of the class variable. It can be understood that the Naïve Bayes Classifier method obtains the optimal performance in our AI modeling. Naïve Bayes Classifier is frequently used for model building in healthcare and can have an optimal performance [32,40,48]. Finally, we implemented the Naïve Bayes Classifier model in practice.

To the best of our knowledge, this is the first AI research aimed at lung resection surgery. Previous studies have provided encouraging information for the development of AI techniques used for medicinal prediction, such as the prediction of postoperative outcomes, mortality rate for mechanically ventilated patients, and the possibility of extubation in intensive care units [28,38,49]. AI approaches were introduced for faster risk evaluation than traditional approaches and were more effective via digitalization. A better prediction model may help improve physician-patient communication and medical settings.

## 5. Limitations

First, since this was a single-hospital study, our results might not be generalizable to other hospitals. Second, the new reversal agent for muscle relaxants used in this study is a self-paid medication in Taiwan, and not all patients are willing to pay for this. Third, according to anesthesia textbooks, ppoFEV1 and ppoDLCO have been established to be positively correlated with the ability to wean immediately after lung surgery. We used ppoFEV1 and ppoDLCO from the Chi Mei Medical Center’s big data to set up the models. However, in actual applications, post-OP data are not available before surgery in the pre-anesthetic clinic, because the actual resection lung volume must be determined by the thoracic surgeon at the time of the operation. Fortunately, anesthesiologists can estimate ppoFEV1 and ppoDLCO, using pre-operative FEV1, pre-operative DLCO, and chest computed tomography reports, which were available in the pre-anesthetic clinic. Anesthesiologists can also immediately consult chest surgeons via phone calls for more information about complex cases.

### Future Research Directions

Although Figure 4 and Figure 5 show that the AI system can improve the communication between anesthesiologists and patients from limited interview questionnaires, a detailed survey of satisfaction requires further research, including analysis of the patients’ age, education level, and previous experience of anesthesia, operation, and mechanical ventilation.

## 6. Conclusions

Pre-anesthetic consultation is a critical step in the pre-operative period, especially for risk assessment if patients cannot wean immediately, requiring high concentration oxygenation and stage weaning in the ICU. Explaining the indication of stage weaning before surgery can greatly reduce the worry of family members and the tension between physicians and patients. Many factors must be considered before weaning the endotracheal tube immediately after the lung resection surgery. We built an AI-assisted prediction system for weaning possibility based on big data and AI algorithms. The NB model performed well according to the model testing report and patient acceptance investigation. We applied the AI-assisted prediction system in our pre-anesthetic evaluation clinic, and continuously collected feedback from physicians and patients for system improvement. We called patients for follow-up studies considering other possible risk factors for model building, including medical image and pre-operation data, as well as extending a variety of other major surgeries that have a high probability of stage weaning of ventilation. A detailed interview questionnaire should be arranged to present the degree of improvement in satisfaction and understanding after AI system application in future studies.

AI is undoubtedly an important technological innovation in medical care today; however, opening the “black box” [50] and strengthening its interpretability is expected to increase acceptance by medical staff to continue.

### Practice Implications

We built an NB algorithm-assisted prediction system for weaning possibility, based on big data of lung resection surgery from Chi Mei Medical Center, and applied the prediction system in our pre-anesthetic evaluation clinic. All of the patients agreed (50% strongly agreed and 50% agreed) that digitalization improved their understanding of the risk of inability to wean immediately, and the demand for high concentration oxygenation and ICU care. Anesthesiologists, both the junior and senior groups, felt that the AI predictive system improved the efficiency of explaining and communicating to patients and the family members, as well as their understanding, during the pre-anesthetic consultation.

## Figures and Tables

**Table 1:** Baseline characteristics of lung resection patients used in machine learning modeling.

Feature	Overall (*n* = 709)	Wean from Ventilator Immediately (*n* = 555)	Cannot Wean Immediately (*n* = 154)	*p*-Value
Postoperative lung function, *n* (%)				
ppoDLCO > 40 and ppoFEV1 > 40	603 (85.05)	523 (94.23)	80 (51.95)	<0.001
ppoDLCO ≤ 40 or ppoFEV1 ≤ 40	106 (14.95)	32 (5.77)	74 (48.05)	
Smoker *, *n* (%)				
Never smoke	468 (66.01)	388 (69.91)	80 (51.95)	<0.001
Had history of smoking	241 (33.99)	167 (30.09)	74 (48.05)	
Desaturation, *n* (%)				
Resting oxygen saturation > 94%	608 (85.75)	505 (90.99)	103 (66.88)	<0.001
Resting oxygen saturation ≤ 94%	101 (14.25)	50 (9.01)	51 (33.12)	
Exercise loading, *n* (%)				
Patient can climb more than 2 floors without resting in between, or can walk for over 6 min	627 (88.43)	526 (94.77)	101 (65.58)	<0.001
Patient can nether climb more than 2 floors without resting in between nor walk for over 6 min	82 (11.57)	29 (5.23)	53 (34.42)	
Severe CAD ^#^, *n* (%)				
No history	623 (87.87)	511 (92.07)	112 (72.73)	<0.001
History of severe CAD	86 (12.13)	44 (7.93)	42 (27.27)	
Major diseases ^, *n* (%)				
No	620 (87.45)	511 (92.07)	109 (70.78)	<0.001
Yes	89 (12.55)	44 (7.93)	45 (29.22)	
Quit smoking before operation, *n* (%)				
Still smoking	573 (80.82)	468 (84.32)	105 (68.18)	<0.001
Quit smoking (cease smoking for more than 4 weeks before lung resection surgery)	136 (19.18)	87 (15.68)	49 (31.82)	
Advanced age, *n* (%)				
<70 years	581 (81.95)	470 (84.68)	111 (72.08)	0.001
≥70 years	128 (18.05)	85 (15.32)	43 (27.92)	
Reversal agent, *n* (%)				
neostigmine	471 (66.43)	341 (61.44)	130 (84.42)	<0.001
sugammadex	238 (33.57)	214 (38.56)	24 (15.58)	

ppoFEV1: Postoperative forced expiratory volume in one second, ppoDLCO: Postoperative diffusing capacity for carbon monoxide; * Smoker: Smoking or not according to the document in the pre-anesthetic risk assessment form, which is recorded by the patient. ^#^ Severe CAD (Coronary artery disease): Patients who had received multiple coronary stent implantation or coronary artery bypass graft surgery, patients who had unstable angina, thallium scan or treadmill showed positive coronary ischemia, or congestive heart failure (left ventricular ejection fraction < 50%). ^ Liver failure, renal failure, morbid obesity (body mass index ≥ 35 Kg/m^2^), or neuromuscular disease that causes weakness such as myasthenia gravis, All the differences between groups were calculated using *t*-test and predetermined analysis with a significance level of *p* < 0.001 (two-sided) using SPSS (Statistical Product and Service Solutions) software (version 19.0, Chicago II., IBM Corp, Armonk, NY, USA).

**Table 2:** Testing results of the predictive models constructed by AI machine learning.

Outcomes and Predictive Models	Accuracy	Sensitivity	Specificity	PPV	NPV	AUC
Wean immediately or not *						
Logistic regression	0.822	0.848	0.814	0.557	0.951	0.908
Random forest	0.779	0.804	0.772	0.493	0.935	0.890
SVM	0.822	0.848	0.814	0.557	0.951	0.856
LightGBM	0.822	0.804	0.826	0.561	0.939	0.908
XGBoost	0.817	0.826	0.814	0.551	0.944	0.909
MLPClassifier	0.831	0.826	0.832	0.576	0.946	0.911
Naive Bayes Classifier	0.845	0.870	0.838	0.597	0.959	0.912

Table 2 lists the testing results of the seven models—logistic regression, random forest, support vector machine [28], Light Gradient Boosting Machine [30], Multilayer Perceptron, Extreme Gradient Boosting, and Naïve Bayes Classifier [32], Wean immediately or not *: whether the patient can wean from the ventilator immediately after lung resection surgery or not. PPV: positive predictive value. NPV: negative predictive value, AUC: Area under receiver operating characteristic curve.

## Data Availability

Since this study is from the data of a single-type operation in a single hospital, in view of the principle of privacy protection, the publicly published data will not contain details of patient data. If readers have research needs, we will provide the original information by request after readers providing relevant IRB approval of their work. We can provide the final import version, in which X values had adapt the threshold in Table S1. The dataset we provide can not to be used without the author’s official approval.

## Supplementary Materials

The following are available online at https://www.mdpi.com/1660-4601/18/5/2713/s1, Table S1: Judgment criteria for binary classification of features, Table S2: Hyperparameter ranges for experiments.

## Abbreviations

AI	artificial intelligence
AUC	area under receiver operating characteristic curve
CAD	coronary artery disease
CT	computed tomography
FEV1	forced expiratory volume in one second
ICU	intensive care unit
Light GBM	Light Gradient Boosting Machine
MLP	Multilayer Perceptron
ppoDLCO	postoperative diffusing capacity for carbon monoxide
ppoFEV1	postoperative forced expiratory volume in one second
ROC curve	receiver operating characteristic curve
SVM	Support Vector Machine
XGBoost	Extreme Gradient Boosting

Cannot wean immediately, or staged weaning: wean the endotracheal tube and mechanical ventilator support after a patient has been transferred to the intensive care unit postoperatively.

Wean immediately: wean the endotracheal tube and mechanical ventilator support immediately after the lung resection surgery was completed, which means the patient could receive postoperative care in the general ward.
