# Supplementary material for: A Real-Time Artificial Intelligence-Assisted System to Predict Weaning from Ventilator Immediately after Lung Resection Surgery
Source: Int J Environ Res Public Health. 2021 Mar 8;18(5):2713. doi: 10.3390/ijerph18052713 (PMC7967444; doi:10.3390/ijerph18052713)
Supplement: Supplementary file 1 [file ijerph-18-02713-s001.pdf]

Table S1. Judgment criteria for binary classification of feature.

	Feature	0	1
1	Estimated postoperative lung function	Estimated ppoDLCO >40 and ppoFEV1 >40	Estimated ppoDLCO <40 or ppoFEV1 <40
2	Severe CAD	None	Patients who had received multiple coronary stent implantation or coronary artery bypass graft surgery before, patients who had unstable angina, thallium patients in whom scan or treadmill showed positive coronary ischemia, or congestive heart failure
3	Exercise loading	Patient can climb more than 2 floors without resting in between, or can walk for over 6 minutes	Patient can nether climb more than 2 floors without resting in between nor walk for over 6 minutes
4	Desaturation: current balance of oxygen supply and demand	Current resting oxygen saturation >94% under room air	Current resting oxygen saturation ≦94% under room air
5	Major diseases	Non	Liver failure, renal failure, morbid obesity (body mass index ≥ 35 Kg/m^2), or neuromuscular disease that causes weakness such as myasthenia gravis
6	History of smoking	Never	Had history of smoking
7	Smoking cessation	Never smoking, or cease smoking for more than 4 weeks before lung resection surgery	Still smoking before lung resection surgery
8	Current age	<70 years	≧70 years
9	Muscle relaxant reversal agent used	neostigmine	sugammadex

Table S2. Hyper-parameters range for experiments.

Method and Hyper-parameter	Values
Logistic regression	
penalty	l1, l2
C	0.001,.009,0.01,.09, 1, 1.2, 5, 15
max_iter	10, 30, 50, 100, 1000
Random forest	
n_estimators	250, 300, 500, 700, 750
max_depth	15, 30, 50
min_samples_split	2, 4, 6, 10
SVM	
kernel	rbf, linear
gamma	scale, 1e-3, 1e-4
C	1, 2, 5, 10, 100, 1000
decision_function_shape	ovo, ovr
shrinking	True, False
LightGBM	
learning_rate	1e-4, 1e-3, 1e-2
num_iterations	200, 500, 700, 950

max_depth	12, 15, 30, 50
random_state	8, 16, 42
XGBoost	
learning_rate	1e-4, 1e-3, 1e-2
gamma	1e-2, 1e-3, 1e-4, 1e-5
num_iterations	200, 500, 700, 950
max_depth	15, 30, 50
num_parallel_tree	2, 5
MLPClassifier	
hidden_layer_sizes	(100), (100,55), (90,60), (200,150,50)
earning_rate_init	1e-2, 1e-3
early_stopping	True, False
Naïve Bayes Classifier	
var_smoothing	2e-9, 1e-09, 1e-06, 1e-04, 1e-02, 1e-01, 0.2, 0.25, 0.3, 0.35

The hyper-parameters that are not described in this table are set to the default values used in the scikit-learn library.
